# An Observational Study of Heart Rate Variability Using Wearable Sensors Provides a Target for Therapeutic Monitoring of Autonomic Dysregulation in Patients with Rett Syndrome

**DOI:** 10.3390/biomedicines10071684

**Published:** 2022-07-13

**Authors:** Jatinder Singh, Shashidhar Ameenpur, Ruksana Ahmed, Salah Basheer, Samiya Chishti, Rosie Lawrence, Federico Fiori, Paramala Santosh

**Affiliations:** 1 Department of Child and Adolescent Psychiatry, Institute of Psychiatry, Psychology and Neuroscience, King’s College London, London SE5 8AF, UK; jatinder.singh@kcl.ac.uk (J.S.); shashidhar.ameen@slam.nhs.uk (S.A.); salah.basheer@slam.nhs.uk (S.B.); samiya.chishti@slam.nhs.uk (S.C.); rosie.lawrence@slam.nhs.uk (R.L.); federico.fiori@kcl.ac.uk (F.F.); 2 Centre for Interventional Paediatric Psychopharmacology and Rare Diseases (CIPPRD), South London and Maudsley NHS Foundation Trust, London SE5 8AZ, UK; ruksana.ahmed@slam.nhs.uk; 3 Centre for Interventional Paediatric Psychopharmacology (CIPP) Rett Centre, Institute of Psychiatry, Psychology and Neuroscience, King’s College London and South London and Maudsley NHS Foundation Trust, London SE5 8AZ, UK

**Keywords:** heart rate variability, autonomic indices, autonomic dysregulation, Rett Syndrome

## Abstract

Rett Syndrome (RTT) is a complex neurodevelopmental disorder that has multi-system involvement with co-occurring epilepsy, breathing problems and autonomic dysregulation. Autonomic dysregulation can increase the risk of cardiorespiratory vulnerability in this patient group. Assessment of heart rate variability (HRV) provides an overview of autonomic health in RTT and offers insight into how the sympathetic and parasympathetic components of the nervous system function. However, to our knowledge, no study has evaluated HRV in Rett patients to assess how the dynamics of autonomic function vary with age and changes during the day and/or night. Using non-invasive wearable sensors, we measured HRV in 45 patients with RTT and examined the time and frequency domain sympathetic and parasympathetic indices. Among the HRV indices assessed, heart rate decreases with age and is lower in the night across all ages studied. The sympathetic index (SDNN) and the parasympathetic indices (RMSSD and pNN50) are not seen to change with age. Nevertheless, these indices were all higher during the day when compared to the night. Our findings appear to show that Rett patients are less adaptable to autonomic changes during the night. In the clinical setting, this might be more relevant for patients with severe psychopathology.

## 1. Introduction

Heart rate variability (HRV), also known as the time variation in the beat-to-beat interval (RR interval), captures the functioning of the autonomic nervous system (ANS). The variation is due to the autonomic control of the sinoatrial node that provides an instant snapshot of changes in heart rhythm [1]. When viewed holistically, HRV is tightly regulated by the central autonomic network (CAN) [2] and involves an overlap of neuronal networks within the brainstem, prefrontal cortex, limbic system and other higher-order brain structures [3]. Patterns in sympathetic and parasympathetic responses can represent changes in HRV, and the sympathovagal balance provides an insight into the vagal tone [4]. These responses are bidirectional between brain structures involved in autonomic control, suggesting that HRV is a valuable marker for CAN integration [5].

Studies on sympathovagal balance have used indices for HRV to explore differences in the physiological responses under the control of the sympathetic nervous system (SNS) and the parasympathetic nervous system (PNS) [6]. These indices can be categorised into frequency domain, time domain and non-linear parameters [7]. Non-linear methods of HRV have not gained traction when compared to frequency and time domain methods [8]. Useful time domain indices are RMSSD (root mean square of successive differences), SDNN (standard deviation of all NN intervals) and pNN50 (percentage of successive R-R intervals that differ by more than 50 ms) [9]. Some indices, such as RMSSD and pNN50, are indicative of vagally mediated (i.e., parasympathetic) HRV [7,9,10], while the SDNN reflects both the parasympathetic and sympathetic components of the ANS [7,9] and is a measure of ANS flexibility. A common frequency domain index is the low-frequency (LF) and high-frequency (HF) ratio (LF/HF) that provides insight into the sympathovagal balance [11,12].

In recent years, studies have shown that measurements of HRV can improve clinical outcomes in some patient groups. A systematic review with a meta-analysis has suggested that HRV can be used as a biomarker to assist in accurately monitoring functional outcomes in individuals following acquired brain injury [13]. There are also associations between HRV and electrodermal activity (EDA) in those with complex neuropsychiatric disorders such as aggression and anti-social spectrum disorders [14]. Vagal activity was also used as a proxy biomarker of disease severity in individuals with schizophrenia and bipolar disorder [15]. HRV parameters can also help unravel patterns of autonomic dysregulation in other neurological disorders. A random-effects meta-analysis for HRV parameters showed lower parasympathetic activity in individuals with post-traumatic stress disorder (PTSD), and a higher LF/HF ratio was noted for those in the PTSD group when compared to controls [16]. Other observations suggest that individuals with autism spectrum disorder (ASD) have a defective vagal brake with concomitant autonomic inflexibility to different stimuli. This autonomic hyperarousal in ASD is thought to exacerbate the emotional and behavioural problems typically seen in these individuals [17]. However, some others have hypothesised that autonomic function is not abnormal in those with ASD [18]. Nevertheless, when viewed more broadly with other neuropsychiatric disorders, the general view is that HRV is lower in those with a diagnosed mental illness when compared to healthy individuals [19]. Interestingly, HRV parameters also remained depressed in individuals that were also medication-free [19].

When viewed through the lens of Rett Syndrome (RTT), a highly impairing paediatric neurodevelopmental disorder, parallels can be drawn from studies on HRV, especially from the viewpoint of a defective CAN where brainstem structures could be compromised. Our previous evidence synthesis has suggested that in patients with RTT, brainstem immaturity, particularly in those areas responsible for the regulation of CAN such as the Kölliker-Fuse and nuclei of the solitary tract, leads to an impaired autonomic tone, increasing the susceptibility of patients to cardiorespiratory complications [20]. Moreover, in a pilot study, we have previously used non-invasive wearable sensors to profile emotional, behavioural and autonomic dysregulation (EBAD) in 10 Rett patients to assess whether biomarkers of HRV and electrodermal activity (EDA) can be used in the management of EBAD in these patients [21]. Physiological biomarkers measured by wearable sensors can also help to demarcate patients with RTT from those with ASD [22].

In patients with RTT, autonomic dysregulation is a significant clinical problem (for a systematic review, see [20]). However, minimal attention has been placed on the different HRV indices in Rett patients and the impact these could have on understanding autonomic health in Rett patients across the age range. We have previously surmised that the defective neurobiological pathways underpinning autonomic dysregulation at birth have probably already been consolidated [23]. It is likely that given the differences in autonomic profiles seen in Rett patients [24], the autonomic dysregulation in RTT follows a non-linear trajectory. However, there is minimal information on how the dynamics of this dysregulation change across the age range in Rett patients and whether this changes between day and/or night. Under these circumstances, it would be sensible to explore HRV metrics in patients with RTT to better understand whether some patients may be more at risk of autonomic inflexibility that could precipitate into a deleterious cardiorespiratory event. The purpose of this observational study was, therefore, to: (I) investigate the autonomic features of HRV in patients with RTT, (II) determine whether any patterns emerge for individual HRV indices across the age range and (III) to apply this knowledge to gain insight into the current management of autonomic dysregulation (and EBAD) in patients with RTT. A greater understanding of autonomic health and HRV dynamics in individuals with RTT would also help in guiding interventions to assist in managing the adverse consequences that autonomic dysregulation might have on immature brain structures in the developing Rett brain.

## 2. Materials and Methods

### 2.1. Study Participants

All 45 subjects were recruited from the Centre for Interventional Paediatric Psychopharmacology (CIPP) Rett Centre, and either had a historical diagnosis of RTT based upon their genetic mutation or a clinical diagnosis [25] that a Consultant Child and Adolescent Psychiatrist confirmed. The co-occurring diagnoses from each subject were obtained from medical notes.

### 2.2. Heart Rate Variability Measurements

Heart rate variability (HRV) measurements were taken at day and night using the Empatica E4 wearable sensor device [26]. While this device allows the capture of HRV indices, EDA and movement data, for this observational study, only blood volume pulse (BVP) was utilised to determine the HRV indices. The HRV data were separately evaluated at day and night for each subject. The parents were trained in the use of the E4 device and ensured that the device was on during the period of data capture.

### 2.3. Assessment of Different Autonomic Indices

The different autonomic indices (mean HR (ms), SDNN (ms), RMSSD (ms), pNN50 (%), LF (nu), HF (nu) and the LF/HF ratio) were determined using the methodology previously described for Rett patients [21]. Briefly, data from the E4 devices were uploaded to the Empatica Cloud using Empatica Manager Software. HRV was determined using the E4 device internal algorithm to obtain the inter-beat interval (IBI) data from raw BVP signals that were captured for each subject using the E4 device. This internal algorithm removes artefacts caused by noise in the BVP signal [27]. For HRV analyses, Kubios software (Kubios HRV Premium, version 3.5.0) was used to estimate the individual HRV indices for each subject. Artefacts in the IBI time series were managed using the validated Kubios artefact correction algorithms [28]. A medium noise level correction and automatic beat correction filter were applied. Each patient had their HRV indices presented as a report in a PDF format. This relies on the recording periods and the proportion with IBI signals, and for each subject is presented in Supplementary Information S1. The mean (minimum and maximum) recording length data (hours, minutes, seconds) analysed during the day for HRV were 04:50:58 (00:09:21; 19:02:43) (*n* = 45). The mean IBI captured was 37%. During the night, the mean recording length data for HRV were 08:39:22 (04:33:44; 12:42:01) (*n* = 43), with a mean IBI capture of 80%. Seven subjects with values (%) of IBI less than 10% were excluded in the day and night comparisons to reduce differences in data length and increase the accuracy of the HRV comparison. For this comparison, 36 subjects were used, and the length of daytime data recording, mean (minimum and maximum), was 04:54:31 (01:21:06; 13:20:00), with a mean IBI capture of 41%, and for the night was 08:47:51 (04:33:44; 11:54:16), with a mean IBI capture of 81%.

### 2.4. Data Management and Statistical Analyses

To extract data from the Kubios HRV Premium PDF file, ABBYY Fine Reader (version 15) was used. To do this, ABBYY Fine Reader with Optical Character Recognition (OCR) reading software was used to open the Kubios PDF report. Tables within the PDF report were recognised by the OCR software; however, if tables were not recognised, table lines were formatted as needed. Once the tables were reformatted, the tables were exported into an Excel document (Microsoft Excel, 2019). For data analysis, R Studio (version 4.1.1) was used. Readxl [29], Tidyverse [30], GGPlot2 and Psych packages were used to transform the data. To import an Excel datasheet into R Studio, the readxl package (version 1.4.0 [29]) was used. Tidyverse package (version 1.3.1 [30]) was used to clean data, apply filters, remove data from tables and add data to tables. To calculate the mean, median, maximum, minimum and standard deviation, Psych package (version 2.2.5 [31]) was utilised. R-squared values were determined by writing a function using R Studio. Finally, the GGPlot2 package (version 3.3.6 [32]) was used to produce the graphs and present the 95% confidence intervals. The *t*-test was used to assess the differences between the day and night HRV indices. Statistical significance was set at *p* values of <0.05.

## 3. Results

### 3.1. Subject Demographics

In our observational study, 45 subjects were included (44 female and 1 male). The characteristics of each subject are presented in Table 1 (mean age (±SD, min; max): 16.46 years ± 9.29 (minimum age: 3 years and 1 month; maximum age: 41 years and 1 month)). The mutational profile was documented for 30 subjects. For 13 subjects, the mutational profile was unknown. Atypical RTT was noted for one subject and one subject had a clinical diagnosis. Epilepsy was the most common comorbid physical illness, found in 47% of subjects, followed by constipation (27%) and dystonia (16%). The frequency of GERD (Gastroesophageal Reflux Disease) and autonomic dysregulation was the same (11%). Amongst the co-occurring neuropsychiatric diagnoses, generalised anxiety disorder (GAD) and autism spectrum disorder (ASD) were found in approximately 18% of the subject population, followed by attention deficit hyperactivity disorder (ADHD) (11%).

### 3.2. Individual Characteristics of HRV Indices

The mean heart rate (HR) alongside time domain indices (SDNN, RMSSD and pNN50) and the frequency domain index (LF/HF ratio) for each subject are presented in Supplementary Information S2. The measurements for each subject are shown for both day and night. Two subjects were noted as having only daytime recordings. There were quite prominent shifts between the day and night-time values for HR among subjects. This inter-subject variability was also mirrored for the time domain indices as well as in sympathovagal imbalance (reflected by the LF/HF ratio), which ranged from 0.23 to 6.86 across the patient population.

### 3.3. HRV Indices across the Age Range during the Day and Night

#### 3.3.1. HR

When looking at the group HRV measurements across the age range, overall, the pattern suggests a decrease in HR across the age range with a concomitant trend of higher HR during the daytime compared to night-time values (Table 2). The highest mean HR values were noted for subjects less than 5 years of age. 

#### 3.3.2. SDNN, RMSSD and pNN50

For the time domain indices, the results suggest that during the day, there is an upwards trajectory for SDNN between 5 and 10 years of age with a plateauing. However, this trend is not so evident during the night. Similarly, for RMSSD and pNN50, there was a modest increase from 5 to 10 years of age and then a levelling off from >10 years during the day. During the night, while there was no noticeable difference for RMSSD values with age, subjects < 5 years had lower night-time pNN50 values when compared to the other age groups (Table 2B).

#### 3.3.3. LF, HF and LF/HF 

The LF component of HRV was stable across the age range during the day (Table 2A). This was mirrored by night-time LF values. Similarly, HF values did not change appreciably with age during the day but did show some inclination to be lower during the night for Rett patients less than 5 years of age when viewed with daytime values of the same age (Table 2B). The LF/HF ratio values also remained stable across the different age ranges during the day (Table 2A). In comparison, night-time values appeared to be elevated for some age groups, especially in those less than 5 years of age and 16–20 years of age (Table 2B). Despite these descriptive trends, overall, daytime LF, HF and LF/HF values were not statistically significant when compared to night-time values.

### 3.4. Comparisons between Day and Night for HRV Indices

When day and night-time HRV indices were compared (*n* = 36) across the entire age range, mean HR, SDNN, RMSSD and pNN50 were all statistically significant (values ranged between *p* = 0.040 and *p* < 0.001) (Table 3). In contrast, day and night-time LF and HF components of HRV, and the LF/HF ratio, were not considered significant (*p* > 0.05). The largest difference existed for day and night-time mean HR, as evidenced by the highest t value, followed by t values for SDNN and RMSSD. Differences between day and night-time values for individual patients (*n* = 43) are shown in Supplementary Information S3.

### 3.5. Scatter Plots for HRV Indices

**Figure 1:**
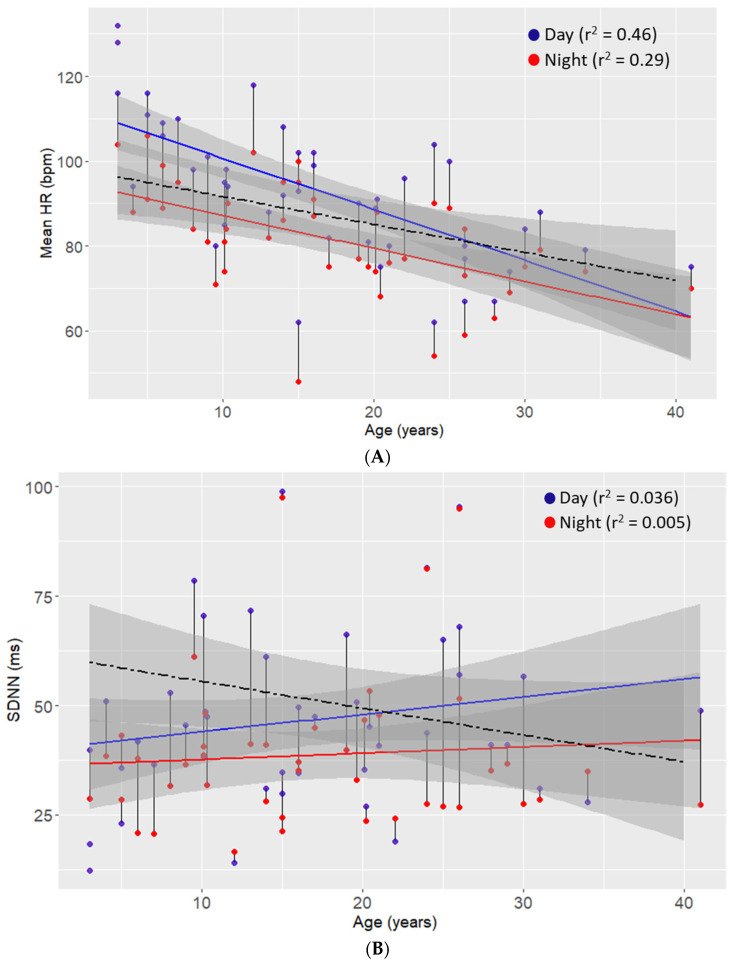

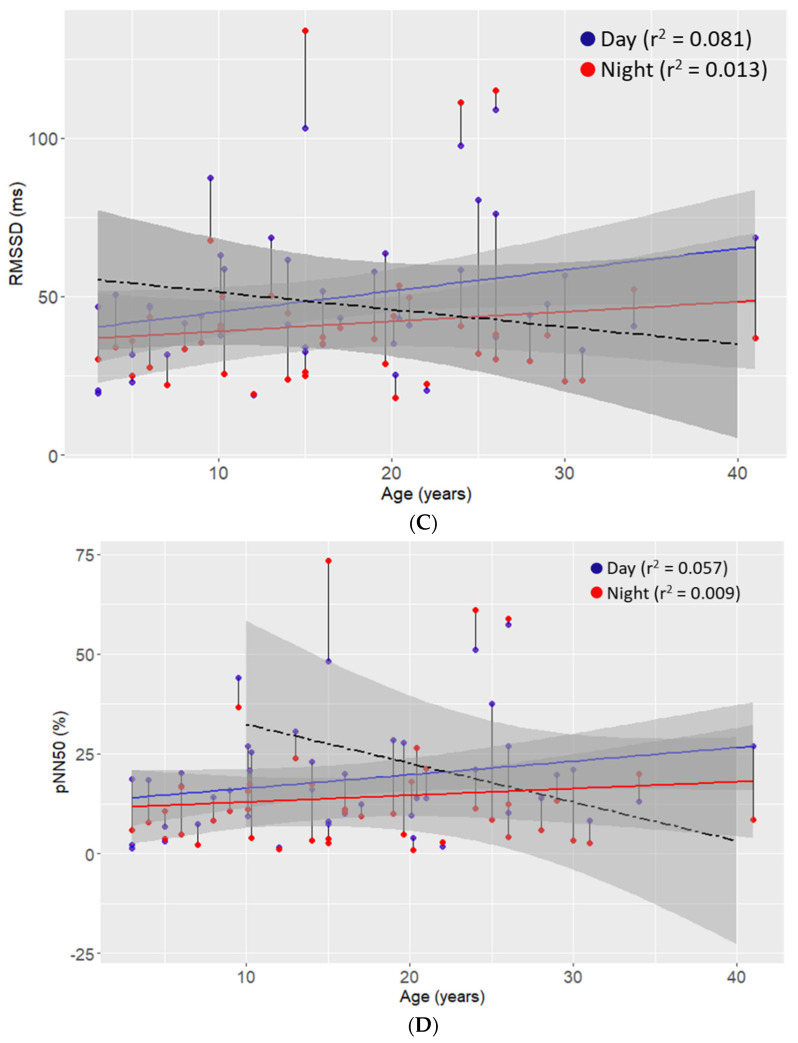

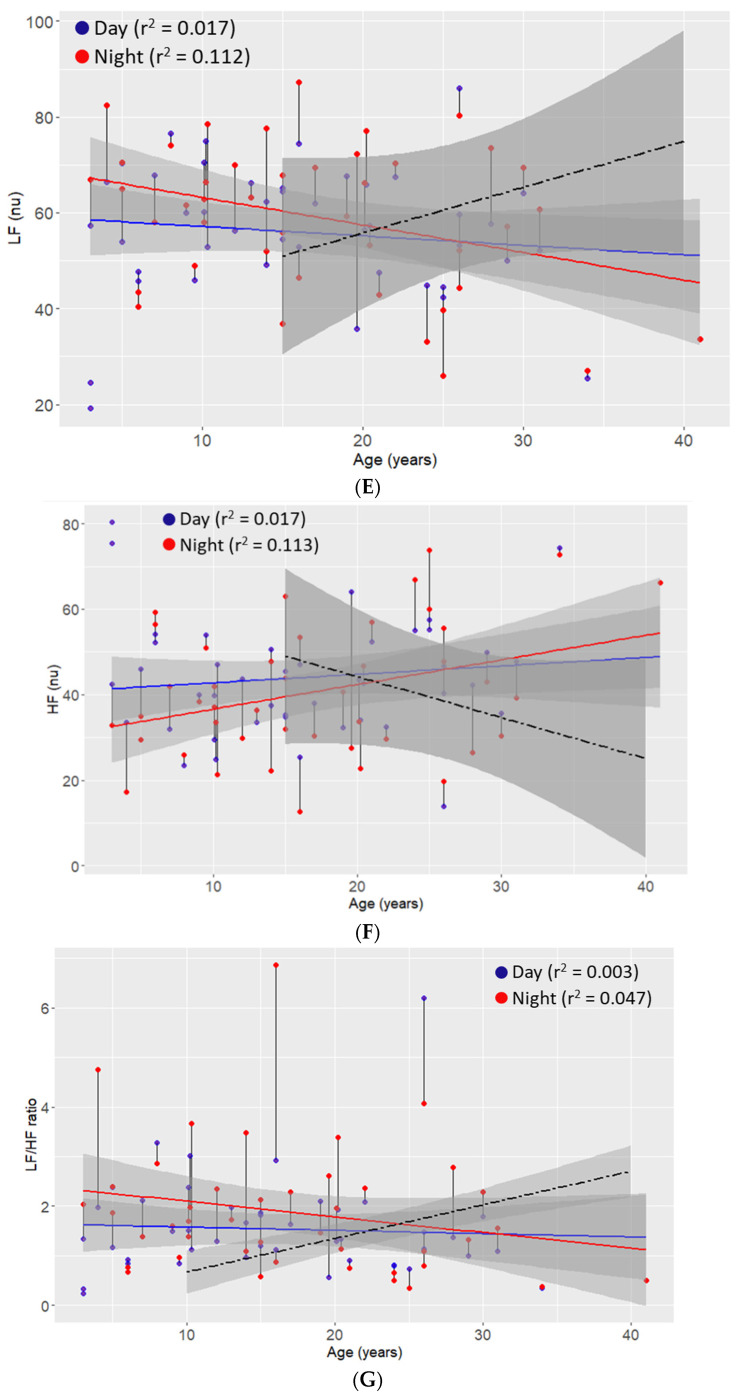
Scatter plots for the different HRV indices ((**A**): Mean HR; (**B**): SDNN; (**C**): RMSSD; (**D**): pNN50; (**E**): LF; (**F**): HF and (**G**): LF/HF ratio). Abbreviations: bpm (beats per minute); HF (high frequency); HR (heart rate); LF (low frequency); LF/HF (low frequency/high frequency); Med (median), Min (minimum); Max (maximum); ms (millisecond); nu (normalised units); pNN50 (percentage of successive R-R intervals that differ by more than 50 ms); RMSSD (root mean square of successive differences); SD (standard deviation); SDNN (standard deviation of all NN intervals). Notes: Lines are presented with 95% confidence intervals, and where indicated, norms are presented as a dashed line (see also Supplementary Information S4).

Scatter plots for all HRV indices with age are presented in Figure 1A–G and allowed a deeper insight into age-related changes in HRV indices. For each index, normal values according to age range are also provided (see also Supplementary Information S4 for a table of reference values).

#### 3.5.1. HR

As in the normal values, mean HR decreases with age (Figure 1A). Compared to norms, for Rett patients, this association is modest but appears to be more prominent during the day, especially for the younger age ranges. The association between HR and age also seems to be more robust during the day (r^2^ = 0.46) when compared to the night (r^2^ = 0.29), and this difference was considered statistically significant (t = 10.18, df = 35, *p* < 0.001). 

#### 3.5.2. SDNN, RMSSD and pNN50

The scatter plots for SDNN (Figure 1B), RMSSD (Figure 1C) and pNN50 (Figure 1D) showed no obvious trend for age-related changes. However, the daytime measurements for these time domain indices were consistently higher across the different age groups, as evidenced by the statistical significance between night and day for SDNN (t = 3.682, df = 35, *p* < 0.001), RMSSD (t = 2.899, df = 35, *p* < 0.001) and pNN50 (t = 2.124, df = 35, *p* = 0.040). The magnitude of this effect was greater for both SDNN and RMSSD when compared to pNN50.

#### 3.5.3. LF, HF and LF/HF

There was no association between day and night-time R^2^ values for the LF (t = −1.074, df = 35, *p* = 0.289) and HF (t = 1.079, df = 35, *p* = 0.287) components of the HRV (Figure 1E,F, Table 3). Night-time LF/HF values appeared to be higher, especially for some age groups, however, this was not significant (t = −1.574, df = 35, *p* = 0.124) and suggests that there is no association between day (r^2^ = 0.003) and night-time (r^2^ = 0.047) LF/HF values and age (Figure 1G). Both day and night-time LF/HF values also showed a departure from norms.

## 4. Discussion

The purpose of our observational study was to: (I) investigate the autonomic characteristics in Rett patients, (II) assess whether any day and night-time trends emerge and (III) assess whether the findings enrich our current understanding of autonomic dysregulation. The mean HR decreased with age in Rett subjects, and this association seemed to be more robust during the day. Mean HR was also statistically significant during the day when compared to the night across the entire age range in Rett patients. In the normal population, HR declines linearly with age [33], and this drop is thought to be caused by age-dependent depression in cardiac pacemaker activity [34,35]. During the period of a reduction in maximum HR with age, there is also an augmentation in the sympathetic drive [34]. Similar to the normal population, in Rett patients, HR also appears to be developmentally driven. The younger age ranges have a much larger inflexion in HR between the day and night-time epochs. The inherent autonomic dysregulation alongside the complex symptom pattern seen in Rett patients probably magnifies the typical age-related autonomic effects on HR. This finding is important because it suggests that Rett patients have a bimodal day and night autonomic functioning pattern. Higher HRV reflects autonomic flexibility, which is suggested to lead to better psychological outcomes [36]. In the context of Rett patients, younger patients may be more prone to having large fluctuations in HRV between the day and night. The resulting night-time autonomic inflexibility due to depressed HR can trigger stressors that could worsen EBAD symptoms that might otherwise have been more easily detected during the day. Disturbances in breathing and HR rhythms occur both during the day and night in Rett patients [37], and some others have suggested that poor sleep hygiene was more prevalent in age groups between 20 and 30 and >30 years of age [38]. When viewed together with the results from our study, night-time autonomic inflexibility would make it less likely for Rett patients to adapt to stressors of EBAD. Night-time monitoring of Rett patients would also be important clinically against the backdrop of increased sympathetic activity with age [34], especially in the context of refractory epilepsy. Depressed sympathovagal balance is seen in children with refractory epilepsy, and the vagal tone fluctuates during the night and day [39]. This further supports the notion that night-time represents a clinically vulnerable time epoch for Rett patients that warrants closer monitoring.

In the next section, two important issues will be considered that arose from the findings of our observational study:

(A) What do age-related changes in HRV indices tell us about the trajectory of autonomic dysregulation in Rett patients?
(B) What other factors may influence HRV in Rett patients?

### 4.1. What Do Age-Related Changes in HRV Indices Tell Us about the Trajectory of Autonomic Dysregulation in Rett Patients?

Previous evidence suggests changes in the ANS with age, such as reduced vagal tone and sympathetic dominance [40]. Others have shown that the components of the PNS, i.e., RMSSD and pNN50, follow a U-shaped distribution, while SDNN (indicative of both SNS and PNS function) decreases linearly with age [41]. More recently, the maturation of the ANS across 5 studies in 4820 healthy individuals aged 0.5 to 20 years was explored [42]. This study revealed a cubic trend for PNS function, i.e., an increase during infancy followed by a plateau phase at mid-childhood and a decline in adolescence. In comparison, SNS functioning demonstrated a linear trend. When viewed from the perspective of maturational trajectories, there are indeed differences in PNS and SNS activities across the life span. The findings in Rett patients suggest a slight upwards trend in SDNN, which is statistically significant during the day; however, neither the day nor night SDNN values seem to be age-related. However, both day and night-time SDNN trajectories in Rett patients differ from reference values. There are very limited data on SDNN in Rett patients. As far as we are aware, only one study has presented SDNN data in another population of Rett patients [43]. In Kumar et al.’s study [43], the median SDNN was 39.3 ms in 23 patients. This value is similar to the median night-time SDNN values in the present study but different to the median SDNN daytime values. The differences could be due to the age ranges of Rett patients assessed. In our study, we assessed SDNN across the age range, while in the previous study [43] the mean age of patients was 9.0 years. Nevertheless, the present findings do indicate that in Rett patients, there is elevated SDNN and hence greater adaptability to HRV changes during the day when compared to the night. 

As with SDNN, the present findings also suggest no apparent age-related changes in PNS activity in Rett patients. The indices for PNS assessment were pNN50 and RMSSD. There was some suggestion that these indices increase from 5 to 10 years and then plateau in Rett patients. However, overall, there was no definitive age-related change in PNS indices in our study. In healthy individuals aged 0.5 to 5 years, RMSSD increases steeply to mid/late childhood and then plateaus. A small decrease in RMSSD occurs at about 11 years old and stabilises thereafter [42]. The values for both day and night-time pNN50 values in our study were also higher when compared to a previous study [43]. What is more apparent is that in Rett patients, both pNN50 and RMSSD values show statistically significant differences between the day and night. These indices indicate PNS function and therefore suggest that the vagal tone at night is particularly disrupted. In the clinical context, this finding would be important for patients with more severe breathing problems. In this group, night-time breathing dysregulation would not be sufficiently compensated by the night-time vagal tone. Therefore, these patients may benefit from a more detailed cardiac assessment, as previously described [20]. 

Previous studies have suggested that a reduced LF/HF is associated with a higher mortality risk [44,45]. In our study, LF/HF does not seem to be age-related and was not considered statistically significant between the day and night. This suggests that the sympathovagal balance in Rett patients is abnormal across the age range and does not change significantly as the disorder progresses. This finding agrees with other studies on Rett patients [46,47]. The sympathovagal balance in RTT likely has epochs where it waxes and wanes in response to stressors such as seizures and breathing dysregulation, or treatment. This could suggest a trend for the higher night-time values in LF/HF observed for some age groups in Rett. When looking more broadly at the RTT population, studies examining the LF/HF ratio have been carried out in 7 studies comprised of 246 subjects with group values ranging between 2.43 and 4.2 [20]. Individual values also range from 6 to 10 [48]. In the present study, the LF/HF values for specific age groups were within this range, and there were also some large inflexions between day and night-time LF/HF values for specific Rett individuals.

The meaningfulness of the sympathovagal balance (i.e., LF/HF ratio) in Rett patients ought to be interpreted with caution. Whether the LF/HF ratio provides an accurate picture of ANS dysregulation is under debate [49,50,51]. Some other studies have suggested that SDNN and RMSSD could be surrogates for LF/HF [52]. The LF/HF ratio can also increase while standing [53]. Long-term HRV measurements suggest that LF/HF decreases in part during a lying position [54]. These findings suggest that it is too simplistic to consider LF/HF as a biomarker for autonomic health, and the ratio ought to be viewed with other indices of ANS activity to determine disturbances in vagal tone. The weak contribution of LF to SNS activity [51] also underscores the impact that breathing dysregulation will have on vagal tone. In particular, the HF only reflects vagal tone when breathing frequency is greater than 9 and 24 cycles per minute [55]. This aspect from the perspective of Rett patients will be discussed in the next section.

### 4.2. What Other Factors May Influence HRV in Rett Patients?

The natural HRV that occurs during the breathing cycle is known as respiratory sinus arrhythmia (RSA) [56]. Respiratory frequency and tidal volume on the RSA are said to be independent from cardiac vagal activity [57], and RSA is suggested to be regulated by the nucleus ambiguus [58] found in the lower brainstem. Autonomic indices provide a picture of autonomic health from HRV that detects the functioning of the sinoatrial node, i.e., cardiac vagal activity. These indices, however, may not yield an accurate picture of the influences that breathing may have on HRV. We know that there are different breathing phenotypes in Rett patients [59], including hyperpnoea, apnoea and breath-holding [24]. Even though the autonomic control of breathing is observed in the HRV, it is more than likely that the disordered breathing profile in Rett patients will affect the interpretation of our findings. It would not be possible to control the tidal volume (using pneumotachography) or respiratory frequency in Rett patients. Instead, the RMSSD would be a more suitable index to consider in Rett patients as it is less influenced by respiratory rates [60]. 

Brain stem immaturity contributes to the autonomic dysregulation in RTT [20]. It could be surmised that given the nascent brain stem immaturity in Rett patients, the HRV responses observed in our study might extend to other organ systems not necessarily under cardiac vagal control, for example parasympathetic regulation of the gastrointestinal system. We have shown that gastrointestinal disturbances can be stressors for EBAD in Rett patients [61], and in this context, RMSSD and pNN50 might be helpful in monitoring autonomic changes driven by lower gastrointestinal issues that are primarily due to PNS activity [62].

## 5. Conclusions

The findings of our present study enrich the current evidence base regarding autonomic dysregulation in RTT and offer new insights into SNS and PNS activity across the age range in these patients. Among the HRV indices assessed in Rett patients, HR appeared to be developmentally driven and was lower at night in all age ranges. The autonomic dysregulation in Rett makes it more likely for the normal age-dependent effects of HR to be amplified. The sympathetic index (SDNN) and the parasympathetic indices (RMSSD and pNN50) did not change with age and showed changes from the patterns seen in the typical population. In Rett patients, SDNN, RMSSD and pNN50 were all higher during the day when compared to the night. This suggests that Rett patients are probably less adaptable to autonomic changes during the night, and clinically, this might be more important for patients with severe psychopathology. Closer motoring of night-time autonomic inflexibility would be needed for patients with severe psychopathology, such as those with refractory seizures and more severe breathing profiles. One other strength of our study was that we could show individual data for all subjects. These data revealed considerable heterogeneity but showed outliers that could be highly clinically significant. The HRV data can be used to risk stratify Rett patients with greater autonomic dysregulation, which may have treatment implications. This approach might also allow further investigation of how severe, moderate or mild pathogenic variants [63] might be associated with different autonomic dysregulation profiles.

Detection and interpretation of HRV indices are complex. Recently, machine learning has been used to facilitate the automatic classification and detection of HRV indices in real-time. Since 2019, unsupervised deep learning has been the mainstay for machine learning approaches adopted for HRV classification [64]. This method has allowed the accurate detection and classification of stress signals from bio-signals [65], and by using an E4 Empatica device, others have used BVP, EDA and accelerometer data to classify stress with a 93% accuracy using a neural network algorithm [66]. When looking through the lens of neurodevelopmental disabilities, machine learning approaches have guided researchers with opportunities unique to this population [67]. This allows strategies of machine learning such as feature extraction, model training and evaluation to be adopted that might help to automate classification of HRV indices in patient populations with global developmental delay. In Rett patients, HRV reports could be mined alongside clinical data to develop prediction models to identify those who might me more at risk of night-time autonomic inflexibility among the general RTT population. Using medical health records, this strategy was adopted by others in patients with Fragile X to accurately predict those with Fragile X as early as possible [68]. In conclusion, our study indicates that non-invasive sensors can provide new information regarding the clinical picture of autonomic dysregulation in Rett patients. Mean HR seems to be the more robust measure for monitoring autonomic flexibility but should be used alongside SDNN to assess overall HRV. Vagally mediated HRV indices allow further investigation of the PNS activity and suggest subtle vagally mediated effects in some age groups. Autonomic dysregulation in Rett patients is governed by a variety of factors, and the information presented here should be viewed with the complex symptom profile seen in patients.

## 6. Study Limitations

Our observational study included a sufficiently large sample of Rett patients to make clinically meaningful conclusions. Our sample consisted of different subjects with different ages rather than the same subjects being followed-up longitudinally. For scatter plots, we used reference values [11,42,69,70,71] for the different HRV indices in the typical population (see Supplementary Information S4); however, these normal values may not be reflective of neurotypical individuals, and for some indices, such as pNN50, LF, HF and the LF/HF ratio, we were unable to obtain values for the younger age groups. Moreover, the reference values do not represent autonomic health during the night-time. This makes the comparison between reference values less accurate, and it should be treated with caution. Moreover, the inter-individual variation in our study limits inferences from other studies. Some other work has also suggested improved methods to analyse data, including deciding whether RMSSD measurements are normally distributed and the use of limits of agreements in Bland–Altman plots to make comparisons [72]. We also did not evaluate the effect of treatments on HRV indices. In our study, Rett patients were on different treatment regimens, and this would be a confounding factor when it comes to the interpretation of the results. Further work would be needed to assess the impact of psychopharmacology on autonomic health across the age range in Rett patients.

Other methodological limitations of using wearable sensors have been previously described [21]. The HRV indices were determined by the IBI, which in turn was dependent on the amount and quality of BVP captured by the sensors and the length of recording sessions. See Supplementary Information S1 for the recording length and % of IBI captured for each subject. Despite these limitations, new information on ultra-short-term HRV norms [72] and recommendations for experimental design [55] have provided new ways to improve upon the methodological limitations in our study.

## Figures and Tables

**Table 1:** Subject demographics.

Subject No.	Age (Years)	Mutation ^	Diagnoses								
			Epilepsy	GAD	Dystonia	Scoliosis	Constipation	ASD	ADHD	GERD	Autonomic Dysregulation
1	10.1	*MECP2* c.473C>T-T158M	No	No	No	Yes	No	No	No	No	No
2	3.4	c.1160_*5215del-p.Pro387_Ser486delinsGln	Yes	No	Yes	No	No	No	No	No	Yes
3	15.6	Unknown	No	Yes	No	No	No	Yes	No	No	No
4	19.6	Unknown	Yes	No	No	No	Yes	No	Yes	No	No
5	15.2	*MEPC2* c.674C>G (p.Pro225Arg)	No	Yes	No	No	No	No	No	No	No
6	5.6	*MECP2* heterozygote c.491C>G	Yes	No	No	No	Yes	No	No	No	No
7	21	Clinically diagnosed	No	No	No	No	No	No	No	No	No
8	11.5	c.455>G (p.Pro152Arg) in exon 4 *MECP2*	No	No	No	No	No	No	No	No	Yes
9	24.6	Partial deletion of exon 4 of *MECP2* gene	Yes	No	No	Yes	No	Yes	No	Yes	No
10	23.7	c.301C>T (p.Pro101Ser)	Yes	Yes	Yes	No	Yes	No	No	No	No
11	41.1	Unknown	Yes	No	No	No	No	No	No	No	No
12	5.5	Heterozygous for *MECP2* c.806delG	No	No	No	No	No	Yes	No	No	Yes
13	10.1	c.808C>T-p.Arg.270	Yes	No	No	No	Yes	No	Yes	Yes	No
14	4.7	Heterozygous c.763C>T p.(Arg225*) in *MECP2*	No	No	No	No	No	No	No	No	No
15	10.3	Heterozygous for the deletion of exons 3i to 4iii* of *MECP2* gene-22kb Xq28 deletion on a CGH with *MECP2*	Yes	No	Yes	No	Yes	No	No	No	No
16	16.2	1157del41	No	No	No	No	No	Yes	Yes	No	No
17	17.11	*MECP2* c.397C>T-R133C	Yes	No	Yes	No	No	No	No	No	No
18	25.8	*MECP2* R306C	No	No	No	No	No	No	No	No	No
19	20.2	*MECP2* c.455C>G-P152R	Yes	No	No	No	No	No	No	No	No
20	13.7	c.422A>C in exon 4 of *MECP2* gene	No	No	No	No	No	No	No	No	No
21	29.6	Unknown	No	No	No	No	No	No	No	No	No
22	28.1	*MECP2*	No	No	No	No	No	No	No	No	No
23	18.8	Unknown	No	No	No	No	Yes	No	No	No	No
24	8.7	Deletions on exons 2 and 4	No	No	No	No	No	Yes	Yes	No	No
25	24.5	Unknown	Yes	Yes	No	No	No	No	No	No	No
26	33.6	Unknown	No	No	No	No	No	No	No	No	No
27	7.3	Heterozygous for c.759_760insT (p.Lys254*) mutation sequence in *MECP2* gene	No	No	No	No	Yes	No	No	Yes	No
28	14.8	General E92 *MEPC2* c.674C>G (p.Pro225Arg)	No	Yes	No	No	No	No	No	No	No
29	14.1	Unknown	No	No	No	Yes	No	No	No	No	No
30	8.1	*MECP2* c.916C>T-R306C	No	No	No	No	No	No	No	No	No
31	12.9	Unknown	Yes	No	No	Yes	No	No	No	No	No
32	9.5	c397T (R113C)	No	Yes	No	No	Yes	Yes	Yes	No	No
33	20.1	Unknown	Yes	No	No	No	No	No	No	No	No
34	4.11	Unknown	Yes	No	No	No	Yes	No	No	No	Yes
35	20.4	R294X in *MECP2*	Yes	No	No	No	No	Yes	No	No	No
36	26.2	Unknown	Yes	No	Yes	No	Yes	No	No	No	No
37	28.7	Unknown	Yes	No	Yes	Yes	Yes	No	No	Yes	No
38	31.1	R168X in *MECP2*	No	Yes	No	No	No	No	No	No	No
39	26.2	*MECP2* c.808C>T-R270*	No	No	No	No	No	No	No	No	No
40	3.4	*MECP2* c.1160_*5215del (p.Pro387_Ser486delinsGln)	Yes	No	No	No	No	No	No	No	Yes
41	10.2	c.799C>T heterozygote, (p.ARG267*)	Yes	Yes	No	Yes	No	Yes	No	Yes	No
42	22.3	*MECP2*	Yes	No	No	Yes	No	No	No	No	No
43	5.2	Atypical Rett syndrome-GABBR-2-related	No	No	No	No	No	No	No	No	No
44	14.8	Heterozygous for the c.880C>T mutation-p.Arg294X	Yes	No	Yes	Yes	Yes	No	No	No	No
45	3.1	*MECP2* 1157del41	No	No	No	No	No	No	No	No	No

Abbreviations: ADHD (attention deficit hyperactivity disorder); ASD (autism spectrum disorder); GAD (generalised anxiety disorder); GERD (gastroesophageal reflux disease); *MECP2* (*methyl-CpG binding protein 2*). Notes: (1) With the exception of subject 22 (male), all subjects were female. (2) ^ Where it is known, the mutation is provided.

**Table 2:** Heart rate variability indices according to age group.

**Day (A)**								
**Age**	***n***	**Mean HR (bpm)** **± SD** **(Med. [Min:Max])**	**Mean SDNN (ms)** **± SD** **(Med. [Min:Max])**	**Mean RMSSD (ms)** **± SD** **(Med. [Min:Max])**	**Mean pNN50 (%) ** **± SD** **(Med. [Min:Max])**	**Mean LF (nu) ** **± SD** **(Med. [Min:Max])**	**Mean HF (nu) ** **± SD** **(Med. [Min:Max])**	**Mean LF/HF Ratio ** **± SD** **(Med. [Min:Max])**
<5	6	116.1 ± 13.4 (116, [94:132])	30.0 ± 14.5 (29.4, [12.3:50.9])	31.9 ± 13.7 (27.3, [19.4:50.6])	8.4 ± 8.09 (4.89, [1.34:18.6])	48.6 ± 21.6 (55.6, [19.2:70.3])	51.2 ± 21.5 (44.2, [29.5:80.3])	1.24 ± 0.86 (1.26, [0.23:2.38])
6–10	10	97.6 ± 9.7 (98, [80:110])	50.2 ± 13.8 (46.5, [36.6:78.6])	50.7 ± 15.8 (46.7, [31.6:87.3])	20.1 ± 10.5 (18.6, [7.4:44.1])	60.2 ± 11.9 (60.0, [45.7:76.5])	39.6 ± 11.8 (39.8, [23.3:54.0])	1.75 ± 0.90 (1.50, [0.84:3.27])
11–15	7	94.7 ± 17.7 (93, [80:110])	48.7 ± 29.6 (34.8, [14:98.9])	51.4 ± 28.5 (41.4, [18.8:103.1])	19.2 ± 16.1 (16.1, [1.6:48.1])	59.7 ± 6.49 (62.3, [49.2:66.3])	40.1 ± 6.45 (37.5, [33.5:50.5])	1.54 ± 0.38 (1.66, [0.97:1.97])
16–20	8	88.6 ± 9.1 (89.5, [75:102])	44.5 ± 12.1 (46.3, [27:66.3])	44.3 ± 12.7 (43.2, [25.2:63.5])	15.7 ± 8.85 (13.0, [3.86:28.4])	59.0 ± 11.6 (59.6, [35.8:74.2])	40.8 ± 11.6 (40.2, [25.4:64.0])	1.61 ± 0.71 (1.48, [0.55:2.92])
>21	14	80.9 ± 12.5 (79.5, [62:104])	51.2 ± 21.0 (46.3, [19:95.3])	57.9 ± 25.5 (52.2, [20.3:108.9])	23.0 ± 15.9 (20.4, [1.76:57.2])	52.0 ± 14.9 (51.1 [25.3:86.0])	47.8 ± 14.9 (48.8 [13.8:74.3])	1.44 ± 1.44 (1.04, [0.34:6.2])
**Night (B)**								
**Age**	***n***	**Mean HR (bpm) ** **± SD** **(med. [min:max])**	**Mean SDNN (ms)** **± SD** **(med. [min:max])**	**Mean RMSSD (ms)** **± SD** **(med. [min:max])**	**Mean pNN50 (%)** **± SD** **(med. [min:max])**	**Mean LF (nu) ** **± SD** **(med. [min:max])**	**Mean HF (nu) ** **± SD** **(med. [min:max])**	**Mean LF/HF Ratio ** **± SD** **(med. [min:max])**
<5	4	97.2 ± 9.06 (97.5, [88:106])	34.6 ± 7.32 (33.5, [28.4:43.1])	31.2 ± 4.82 (32.0, [25:36])	6.98 ± 2.96 (6.8, [3.6:10.6])	71.2 ± 7.83 (68.7, [65.0:82.5])	28.6 ± 7.85 (31.1, [17.3:34.9])	2.76 ± 1.34 (2.21, [1.8:4.7])
6–10	10	84.8 ± 8.74 (84, [71:99])	36.8 ± 12.0 (37.2, [20.6:61.2])	38.6 ± 13.3 (37.5, [22.2:67.6])	12.7 ± 9.99 (10.9, [2.2:36.7])	59.2 ± 12.3 (59.8, [40.3:78.5])	40.6 ± 12.2 (40.0, [21.3:59.2])	1.69 ± 0.94 (1.49, [0.68:3.67])
11–15	7	86.8 ± 18.5 (95, [48:102])	38.5 ± 27.6 (28.1, [16.6:97.6])	46.2 ± 40.3 (26.3, [19.3:133.9])	17.9 ± 25.9 (3.7, [1.03:73.4])	60.5 ± 13.5 (63.1, [36.9:77.6])	39.3 ± 13.5 (36.4, [22.2:63.0])	1.80 ± 0.95 (1.73, [0.58:3.48])
16–20	8	79.3 ± 8.19 (76, [68:91])	39.2 ± 9.15 (38.5, [23.7:53.3])	36.7 ± 10.4 (36.8, [18.1:53.6])	11.3 ± 7.8 (10.1, [0.89:26.4])	66.4 ± 13.1 (67.8, [46.5:87.2])	33.4 ± 13.1 (32.0, [12.7:53.3])	2.57 ± 1.91 (2.12, [0.87:6.86])
>21	14	73.7 ± 10.4 (74.5, [54:90])	40.8 ± 21.7 (31.7, [24.1:94.9])	45.9 ± 29.8 (37.3, [22.3:114.9])	16.7 ± 19.2 (9.9, [2.6:61.3])	50.7 ± 18.1 (48.2, [26.0:80.2])	49.1 ± 18.0 (51.6, [19.7:73.9])	1.38 ± 1.10 (0.94, [0.35:4.07])

Abbreviations: bpm (beats per minute); HF (high frequency); HR (heart rate); LF (low frequency); LF/HF (low frequency/high frequency); Med (median), Min (minimum); Max (maximum); ms (milliseconds); nu (normalised units); pNN50 (percentage of successive R-R intervals that differ by more than 50 ms); RMSSD (root mean square of successive differences); SD (standard deviation); SDNN (standard deviation of all NN intervals).

**Table 3:** Differences between day and night-time HRV indices in the sample population.

Index	Mean	SD	Median	Min.	Max.	*p*-Value	t Value	Degree of Freedom
Mean HR (bpm)	86.69	14.69	88	48	118	<0.001	10.18	35
SDNN (ms)	41.74	16.77	39.2	14	98.9	<0.001	3.682	35
RMSSD (ms)	43.53	20.70	40.4	18.1	133.9	0.006	2.899	35
pNN50 (%)	15.22	13.14	12.33	0.89	73.44	0.040	2.124	35
LF (nu)	59.89	13.58	61.19	25.38	87.24	0.289	−1.074	35
HF (nu)	40.00	13.55	38.76	12.71	74.39	0.287	1.079	35
LF/HF ratio	1.846	1.201	1.579	0.341	6.864	0.124	−1.574	35

Abbreviations: bpm (beats per minute); HF (high frequency); HR (heart rate); LF (low frequency); LF/HF (low frequency/high frequency); Med (median), Min (minimum); Max (maximum); ms (millisecond); nu (normalised units); pNN50 (percentage of successive R-R intervals that differ by more than 50 ms); RMSSD (root mean square of successive differences); SD (standard deviation); SDNN (standard deviation of all NN intervals).

## Data Availability

Data can be obtained upon reasonable request from the corresponding author.

## Supplementary Materials

The following supporting information can be downloaded at: https://www.mdpi.com/article/10.3390/biomedicines10071684/s1, Supplementary Information S1: Recording length and % IBI captured for each subject. Supplementary Information S2: Heart Rate Variability Indices for Each Subject. Supplementary Information S3: Difference between Day and Night Values. Supplementary Information S4: Summary of heart rate variability reference values.
