# Supplementary material for: An Observational Study of Heart Rate Variability Using Wearable Sensors Provides a Target for Therapeutic Monitoring of Autonomic Dysregulation in Patients with Rett Syndrome
Source: Biomedicines. 2022 Jul 13;10(7):1684. doi: 10.3390/biomedicines10071684 (PMC9312701; doi:10.3390/biomedicines10071684)
Supplement: Supplementary file 1 [file biomedicines-10-01684-s001.zip › biomedicines-1769486-supplementary.pdf]

Supplementary Information S1: Recording length and % IBI captured for each subject

Subject No	Recording length*	Percentage IBI	Part of day
1	02:16:19	16	Day
	07:13:25	91	Night
2**	18:04:31	63	Day
3	01:35:15	18	Day
	06:09:28	56	Night
4***	00:09:21	2	Day
	09:08:13	86	Night
5	05:29:51	42	Day
	05:44:47	66	Night
6***	00:37:57	4	Day
	06:38:06	92	Night
7	01:46:11	53	Day
	06:23:40	85	Night
8	11:26:14	74	Day
	08:11:18	90	Night
9***	00:53:49	9	Day
	12:42:01	87	Night
10	03:32:57	46	Day
	10:26:46	65	Night
11	01:51:57	14	Day
	07:01:54	70	Night
12	01:21:06	23	Day
	08:16:14	85	Night
13	04:01:45	35	Day
	09:12:41	76	Night
14	06:18:26	58	Day
	11:00:33	90	Night
15	02:53:48	24	Day
	09:19:05	83	Night
16	03:19:11	37	Day
	07:50:38	92	Night
17	03:45:58	35	Day
	05:02:38	53	Night
18***	00:11:41	8	Day
	08:47:01	73	Night
19	02:39:49	23	Day
	09:42:31	78	Night
20	04:29:09	32	Day

Subject No	Recording length*	Percentage IBI	Part of day
	08:40:15	71	Night
21	05:59:38	40	Day
	08:38:08	96	Night
22	08:42:48	68	Day
	11:54:16	95	Night
23	06:23:46	42	Day
	09:49:14	88	Night
24	01:42:09	13	Day
	09:47:52	89	Night
25	02:31:54	21	Day
	10:07:57	76	Night
26	08:07:36	79	Day
	08:56:47	65	Night
27	04:41:53	39	Day
	10:55:25	85	Night
28***	00:37:19	7	Day
	06:22:05	50	Night
29	05:02:16	36	Day
	08:18:25	90	Night
30	04:24:14	55	Day
	09:39:35	93	Night
31	08:53:10	58	Day
	09:35:33	92	Night
32***	00:47:05	7	Day
	06:56:07	57	Night
33	07:22:36	76	Day
	07:14:26	67	Night
34	04:04:48	23	Day
	05:49:25	88	Night
35	04:28:06	30	Day
	08:31:49	82	Night
36	07:15:43	85	Day
	10:53:45	90	Night
37	02:49:37	24	Day
	10:36:51	85	Night
38	13:20:00	59	Day
	10:54:00	95	Night
39***	01:06:02	7	Day
	04:56:10	56	Night

Subject No	Recording length*	Percentage IBI	Part of day
40**	19:02:43	60	Day
41	07:16:22	57	Day
	10:42:44	78	Night
42	04:45:53	40	Day
	09:25:54	79	Night
43	07:04:01	68	Day
	04:33:44	89	Night
44	02:37:03	15	Day
	10:18:10	78	Night
45	02:21:13	31	Day
	09:42:55	79	Night

Abbreviations: IBI (inter-beat-interval)

Notes:

* Recording length given in hours, minutes and seconds

** Only day-time recordings were available

*** Percentage (%) of IBI less than 10% was excluded from the day and night comparisons

Supplementary Information S2: Heart Rate Variability Indices for Each Subject

Subject No.	Mean HR (bpm)	SDNN (ms)	RMSSD (ms)	pNN50 (%)	LF (nu)	HF (nu)	LF/HF ratio	Recording time
1	95	38.2	37.7	9.34	60.21	39.71	1.516	Day
	81	38.6	40.9	15.65	57.97	41.87	1.385	Night
2*	132	12.3	19.4	2.17	19.24	80.39	0.239	Day
3	99	49.6	51.7	19.94	52.93	46.98	1.127	Day
	91	35.2	37.1	11.09	46.5	53.35	0.872	Night
4	81	50.7	63.5	27.74	35.8	64.05	0.559	Day
	75	32.9	28.9	4.73	72.32	27.63	2.617	Night
5	93	29.8	34	8.03	64.51	35.37	1.824	Day
	100	21.2	26.3	3.77	55.93	43.84	1.276	Night
6	106	41.8	47	20.17	45.73	54.06	0.846	Day
	99	20.8	27.7	4.82	40.35	59.24	0.681	Night
7	80	40.8	41.1	13.99	47.5	52.42	0.906	Day
	76	47.8	49.8	21.3	42.92	56.98	0.753	Night
8	118	14	18.8	1.6	56.18	43.64	1.287	Day
	102	16.6	19.3	1.03	70.01	29.83	2.347	Night
9	100	65	80.6	37.69	42.4	57.48	0.738	Day
	89	26.9	32.1	8.55	26.04	73.9	0.352	Night
10	62	81.4	97.6	50.97	44.94	54.98	0.817	Day
	54	81.3	111.3	61.13	33.11	66.83	0.495	Night
11	75	48.8	68.5	26.94	33.61	66.19	0.508	Day
	70	27.4	36.8	8.45	33.57	66.27	0.507	Night
12	109	41.8	46.5	17.05	47.7	52.2	0.914	Day
	89	37.9	43.7	16.66	43.41	56.4	0.77	Night
13	85	70.6	63.1	26.98	70.44	29.5	2.387	Day
	74	40.6	39.7	11.16	62.88	37.06	1.697	Night
14	111	35.8	31.7	6.82	70.38	29.55	2.381	Day
	91	43.1	36	10.63	70.47	29.47	2.391	Night
15	94	47.5	58.7	25.49	52.85	46.99	1.125	Day
	90	31.9	25.5	3.92	78.55	21.38	3.675	Night
16	102	34.5	34.9	10.08	74.42	25.48	2.921	Day
	87	37.1	35.2	10.38	87.24	12.71	6.864	night
17	82	47.5	43.4	12.33	61.95	38	1.63	Day
	75	44.9	40.2	9.27	69.52	30.41	2.286	Night
18	67	95.3	108.9	57.27	53.2	46.72	1.139	Day
	59	94.9	114.9	58.95	52.15	47.79	1.091	Night
19	91	27	25.2	3.86	65.84	34.11	1.93	Day
	88	23.7	18.1	0.89	77.17	22.8	3.385	Night
20	92	61.1	61.7	23.13	62.38	37.55	1.661	Day

Subject No.	Mean HR (bpm)	SDNN (ms)	RMSSD (ms)	pNN50 (%)	LF (nu)	HF (nu)	LF/HF ratio	Recording time
	86	41	44.9	17.32	51.97	47.82	1.087	Night
21	84	56.6	56.8	21.17	64.12	35.78	1.792	Day
	75	27.6	23.4	3.24	69.51	30.45	2.283	Night
22	67	41.1	44.1	14.01	57.69	42.23	1.366	Day
	63	35.1	29.6	5.87	73.53	26.44	2.781	Night
23	90	66.3	57.8	28.42	67.64	32.29	2.095	Day
	77	39.9	36.6	9.97	59.34	40.61	1.461	Night
24	101	45.6	43.9	15.81	59.92	40	1.498	Day
	81	36.6	35.4	10.73	61.64	38.31	1.609	Night
25	104	43.8	58.3	21.1	44.45	55.27	0.804	Day
	90	27.6	40.6	11.3	39.73	59.96	0.663	Night
26	79	27.9	40.6	12.99	25.38	74.39	0.341	Day
	74	35	52.4	20.1	27.02	72.72	0.372	Night
27	110	36.6	31.6	7.42	67.9	32.04	2.119	Day
	95	20.6	22.2	2.21	57.99	41.93	1.383	Night
28	102	34.8	32.7	7.37	65.16	34.76	1.875	Day
	95	24.4	25	2.63	67.9	31.9	2.128	Night
29	108	31	41.4	16.12	49.22	50.54	0.974	Day
	95	28.1	23.9	3.19	77.66	22.28	3.485	Night
30	98	52.9	41.7	14.21	76.56	23.39	3.273	Day
	84	31.6	33.5	8.34	73.99	25.94	2.853	Night
31	88	71.7	68.5	30.58	66.31	33.59	1.974	Day
	82	41.2	50.4	23.97	63.18	36.48	1.732	Night
32	80	78.6	87.3	44.11	45.84	54.04	0.848	Day
	71	61.2	67.6	36.76	48.97	50.96	0.961	Night
33	89	35.4	35.3	9.55	56.44	43.51	1.297	Day
	74	46.6	43.9	18.08	66.24	33.71	1.965	Night
34	94	50.9	50.6	18.47	66.37	33.51	1.98	Day
	88	38.4	33.9	7.76	82.52	17.34	4.76	Night
35	75	45.1	43	13.83	57.37	42.58	1.347	Day
	68	53.3	53.6	26.48	53.23	46.74	1.139	Night
36	77	57.1	37.2	10.18	86.09	13.88	6.2	Day
	73	51.6	38	12.33	80.26	19.71	4.071	Night
37	74	41	47.6	19.76	50.02	49.9	1.002	Day
	69	36.7	37.9	13.27	57.06	42.91	1.33	Night
38	88	31.1	33.1	8.25	52.2	47.71	1.094	Day
	79	28.4	23.7	2.65	60.75	39.21	1.55	Night
39	80	68	76.2	26.99	59.62	40.23	1.482	Day
	84	26.8	30.3	4.21	44.37	55.55	0.799	Night

Subject No.	Mean HR (bpm)	SDNN (ms)	RMSSD (ms)	pNN50 (%)	LF (nu)	HF (nu)	LF/HF ratio	Recording time
40*	128	18.4	20.3	1.34	24.51	75.34	0.325	Day
41	98	48.6	49.9	20.83	75.02	24.91	3.012	Day
	84	48.3	50	17.39	66.35	33.55	1.978	Night
42	96	19	20.3	1.76	67.42	32.44	2.078	Day
	77	24.1	22.3	2.73	70.29	29.64	2.371	Night
43	116	23.1	23	2.96	53.93	45.98	1.173	Day
	106	28.4	25	3.6	65.03	34.92	1.862	Night
44	62	98.9	103.1	48.13	54.52	45.45	1.2	Day
	48	97.6	133.9	73.44	36.9	63.07	0.585	Night
45	116	39.8	46.8	18.68	57.31	42.5	1.348	Day
	104	28.6	30.2	5.93	67.02	32.81	2.043	Night

Abbreviations:

bpm (Beats Per Minute); HF (High Frequency); HR (Heart Rate); LF (Low Frequency); LF/HF (Low Frequency/High Frequency); ms (millisecond); nu (normalised units); pNN50 (Percentage of successive R-R intervals that differ by more than 50 ms); RMSSD (Root Mean square of Successive Differences); SDNN (Standard Deviation of all NN intervals)

Notes:

*Only day-time recordings were available

Supplementary Information S3: Difference between Day and Night Values

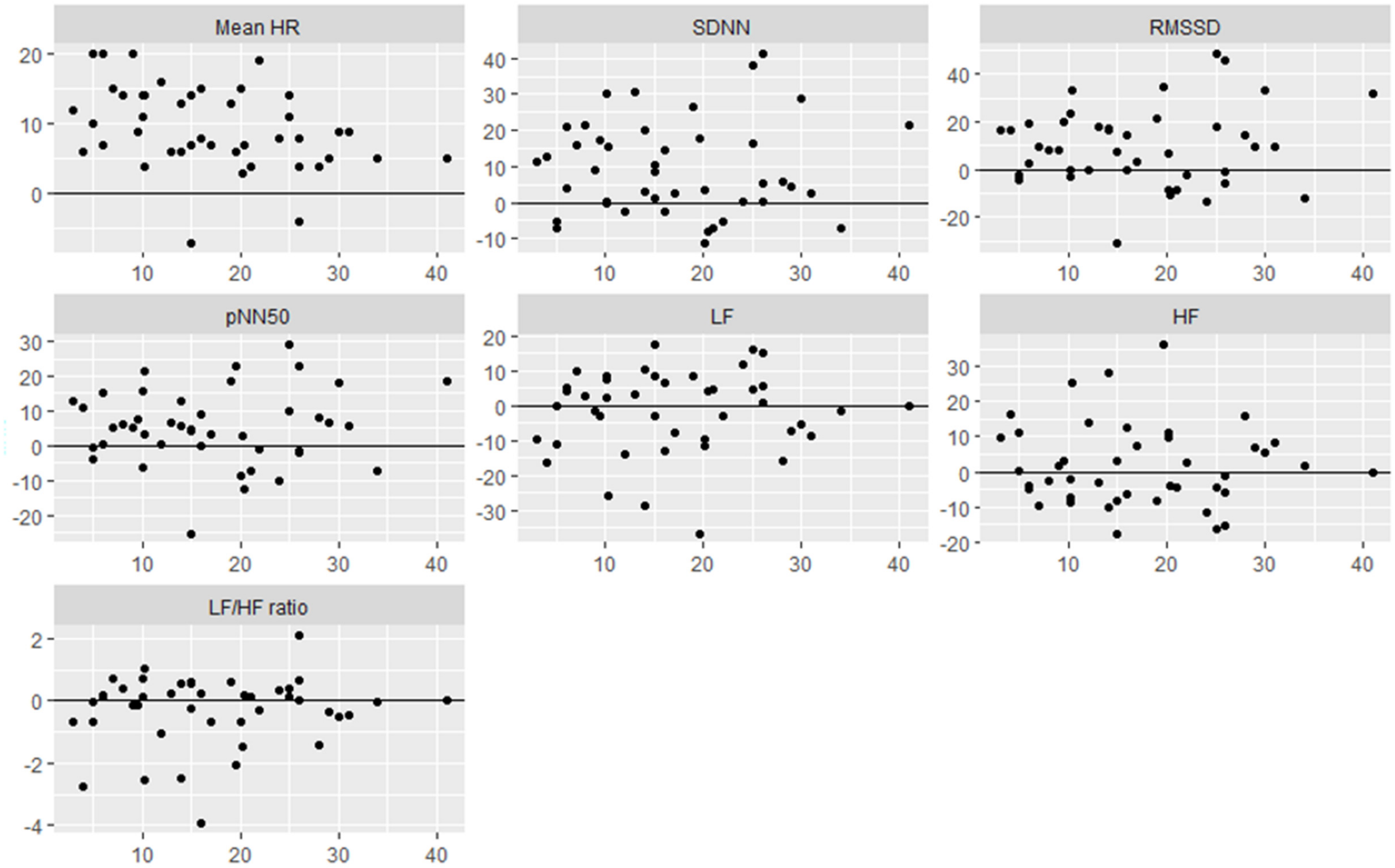

Notes:

Y axis (difference); X axis (age in years)

Supplementary Information S4: Summary of heart rate variability reference values

Age	HR (bpm)	SDNN (ms)	RMSSD (ms)	pNN50 (%)	LF (nu)	HF (nu)	LF/HF ratio	Reference(s)
0.5^	137.9	23.6	11.1	-	-	-	-	2
1-4^	105.1	46.7	34.8	-	-	-	-	2
5^	94.4	60.6	51.6	-	-	-	-	2
5-10^	84 [77–91] (IQR)	60 [42–78] (IQR)	66 [44–91] (IQR)	38 [20–52] (Med)	-	-	0.63 [0.41–1.02] (IQR)	3
12-17^	89.29 (64.4:120.9), (min:max)	66.35 (9.4:193.8), (min:max)	69.00 (12.8:256.3), (min:max)	Not given as %	45.44	54.56	0.83 (0.08:3.79), min:max	4
20-30	80.2 ± 14.8**	46.34* (27.8:77.2)	42.66* (22.3:89.8)	15.06* (3.37;37.56)	61.48*	38.52*	1.60* (0.74:3.63)	1,5
30-40	78.5 ± 15.1**	42.39* (26.0:66.4)	36.50* (20.1:71.1)	11.43* (1.75:31.5)	67.81*	32.19*	2.11* (0.83:4.37)	1,5
40-50	75.3 ± 14.3**	33.71* (20.1:52.9)	30.00* (15.4:53.2)	6.69* (0.79:21.94)	72.26*	27.74*	2.60* (0.85:5.44)	1,5

Abbreviations

bpm (Beats Per Minute); HR (Heart Rate); IQR (Interquartile Range); LF/HF (Low Frequency/High Frequency); Max (Maximum); Med (Median); Min (Minimum); ms (millisecond); pNN50 (Percentage of successive R-R intervals that differ by more than 50 ms); RMSSD (Root Mean square of Successive Differences); SD (Standard Deviation); SDNN (Standard Deviation of all NN intervals)

Notes:

Unless otherwise stated all data is shown for females

^ Values were presented as median

* These are 50th (5th and 95th) percentile values

** Presented as geometric mean HR ± SD for the following age ranges (21-30, 31-40 and 41-50 years of age) in both male and females (Avram *et al.*, 2019)

Where the values are unknown, this is indicated by –

References

[1]. Sammito S, Böckelmann I. Reference values for time- and frequency-domain heart rate variability measures. Heart Rhythm. **2016,** 13, 1309-1316.

[2]. Harteveld LM, Nederend I, Ten Harkel ADJ, Schutte NM, de Rooij SR, Vrijkotte TGM, Oldenhof H, Popma A, Jansen LMC, Suurland J, Swaab H, de Geus EJC; FemNAT-CD collaborators *. Maturation of the Cardiac Autonomic Nervous System Activity in Children and Adolescents. *J. Am. Heart Assoc.* **2021**,*10*, e017405.

[3]. Michels N, Clays E, De Buyzere M, Huybrechts I, Marild S, Vanaelst B, De Henauw S, Sioen I. Determinants and reference values of short-term heart rate variability in children. Eur. J. Appl. Physiol. **2013**, *113*, 1477-1488.

[4]. Sharma VK, Subramanian SK, Arunachalam V, Rajendran R. Heart Rate Variability in Adolescents - Normative Data Stratified by Sex and Physical Activity. *J. Clin. Diagn. Res*. **2015**, *9*, CC08-13.

[5]. Avram R, Tison GH, Aschbacher K, Kuhar P, Vittinghoff E, Butzner M, Runge R, Wu N, Pletcher MJ, Marcus GM, Olgin J. Real-world heart rate norms in the Health eHeart study. *NPJ. Digit. Med.* **2019**, *2*, 58.
